# Investigation and analysis of carbapenem-resistant gram-negative bacterial infection rates across hospitals in Shandong Province in China

**DOI:** 10.3389/fpubh.2022.1014995

**Published:** 2022-11-07

**Authors:** Keke Liu, Hua Xu, Jian Sun, Yuqing Liu, Weiguang Li

**Affiliations:** ^1^Department of Infection Control, Shandong Provincial Hospital Affiliated to Shandong First Medical University, Jinan, China; ^2^Institute of Animal Science and Veterinary Medicine, Shandong Academy of Agricultural Sciences, Jinan, China

**Keywords:** carbapenem resistance bacteria, detection rate, antibacterial drug use, China, hospital infections

## Abstract

**Background:**

The increasing incidence of carbapenem-resistant bacterial infections has become a serious public health threat. This study aimed to investigate and analyze the current regional differences in carbapenem-resistant gram-negative bacteria (CRGN) in a major Province of China, and provide suggestions for preventing hospital infections.

**Methods:**

A questionnaire survey was used to obtain the current data on CRGN from 36 hospitals in Shandong Province, China, from 2019 to 2020. The association between the detection rates and discovery rates of CRGN and the use of antibacterial drugs was analyzed using Spearman's correlation coefficient. In addition, we compared the detection rates of CRGN and antibacterial drugs using hospitals categorized according to different levels and economic areas using the Kruskal-Wallis test.

**Results:**

The average detection rates of CRGN across the 36 hospitals varied from 1.91% to 66.04%. The discovery rate of carbapenem-resistant *Enterobacteriaceae* (CRE) and carbapenem-resistant *Acinetobacter baumannii* (CRAB) remained below 5‰, and that of carbapenem-resistant *Pseudomonas aeruginosa* (CRPA) was below 10‰. Except for CRAB, the correlations between the detection rate and antimicrobial drug use intensity and carbapenem drug use percentage were 0.11–0.29 and 0.31–0.47, respectively. Carbapenem drug use was higher in the provincial hospital group than in the prefecture-level hospitals (*P* < 0.05), and that in the high-economic regional hospital group was higher than in the low-economic regional hospital group (*P* < 0.05).

**Conclusions:**

The detection and discovery rates of CRE were low, and those of CRAB were high in Shandong Province. Larger hospitals have higher carbapenem drug use. These results can be used as a reference for preventing CRGN infections in developing countries and provide a basis for regional carbapenem resistance prevention and control strategies.

## Introduction

The prevalence of multidrug-resistant organisms is a major public health threat that continues to increase globally and is associated with significant morbidity and mortality (1–4). The spread of carbapenem-resistant gram-negative bacteria (CRGN) continues to increase rapidly worldwide, despite considerable efforts in infection prevention and control programs, and targeted interventions for antimicrobial stewardship (5–7). CRGN mainly includes carbapenem-resistant *Enterobacteriaceae* (CRE), carbapenem-resistant *Acinetobacter baumannii* (CRAB), and carbapenem-resistant *Pseudomonas aeruginosa* (CRPA), which pose a significant threat in terms of mortality, medical burden, and trends in antimicrobial resistance (8–11).

Multidrug-resistant organisms harm both infected and non-infected patients. All patients are affected by the lack of appropriate antibiotic regimens due to the subsequent decreased efficiency of antimicrobial agents. Most previous studies (2, 4, 12–14) were laboratory-based and focused on the molecular characteristics and distribution of CRGN. There is a bacterial resistance report of the Chinese mainland published every year (15), but a more detailed regional comparison of the detection rate and carbapenem drug use between hospitals and economic levels is lacking, especially for major provinces.

Shandong Province is an important Province on the eastern coast of China. To the best of our knowledge, there is no currently available regional comparative study on the CRGN infections in Shandong, which prevents the implementation of effective control strategies by local governments. Assessing the association between antibiotic use and antimicrobial resistance could help local physicians and decision-makers better use antibiotics and distribute healthcare funds more effectively, while improving infection control and providing reference strategies for developing countries.

Therefore, this study aimed to compare the detection rate of carbapenem-resistant bacteria and carbapenem drug use at different hospital levels and economic regions of Shandong to provide a reference for developing prevention and control strategies for multidrug-resistant bacteria in different regions.

## Materials and methods

The survey was approved by the participating hospitals and an agreement was signed with each hospital. The survey was anonymous and did not involve the identity information of participants. This study was designed as a retrospective study, and collected data were kept confidentially. Therefore, it was exempt from ethical review.

### Hospital selection and survey content

**Figure 1:**
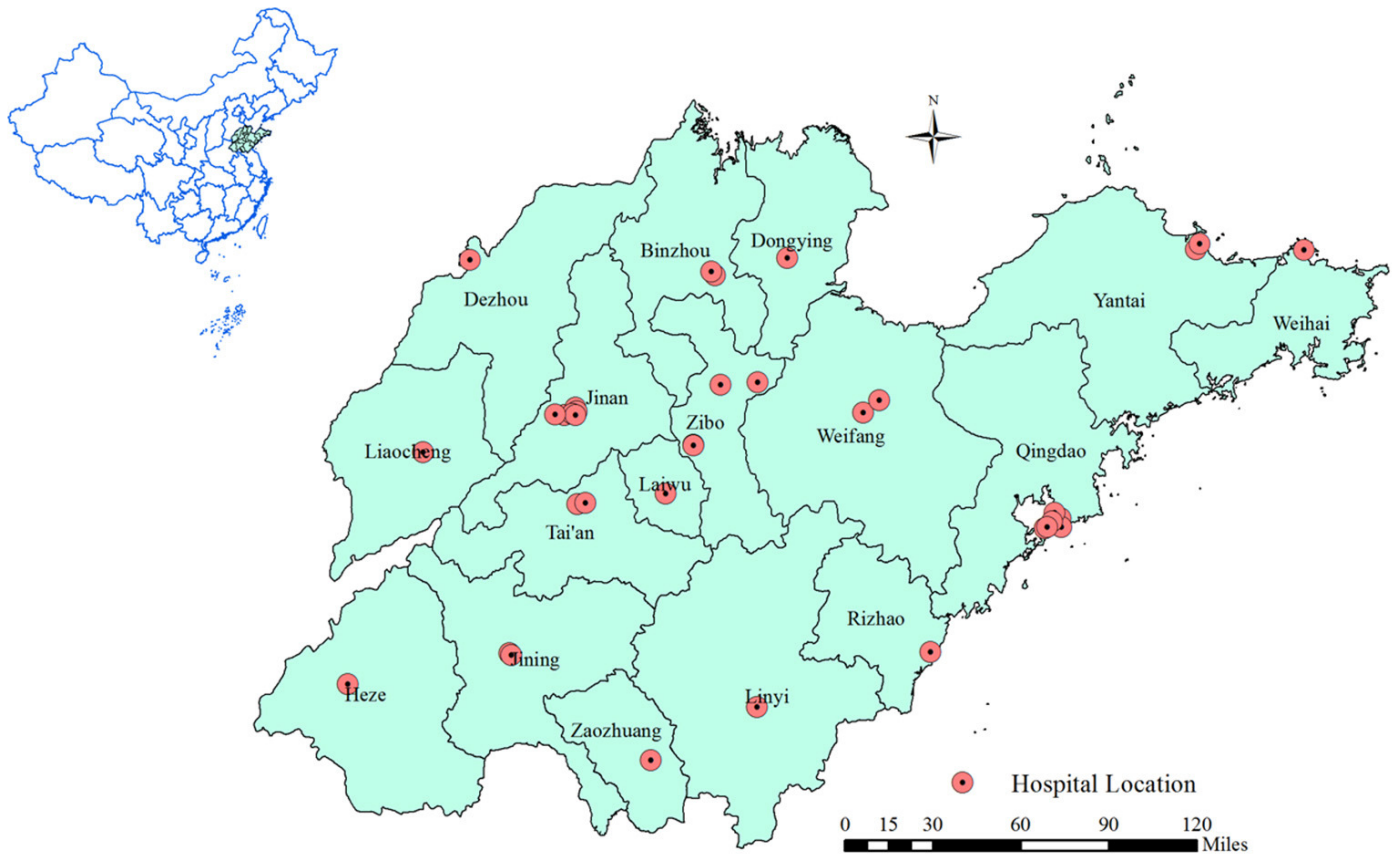
The distribution of 36 hospitals in Shandong Province of China.

Thirty-six representative hospitals were selected in Shandong Province, China (Figure 1). The strains of carbapenem-resistant bacteria and the total number of gram-negative bacteria detected from 2019 to 2020 were investigated using a questionnaire survey. To eliminate copy-strains, during each patient's hospitalization, we assumed that copy-strains would have the same etiological results (including drug susceptibility), and only one result was used from copy-strains for analysis. Detected strains were obtained from various screening samples including throat and rectal swabs, blood samples, and other body fluid samples in this study. In addition, we also collected data on the detection rate of carbapenem-resistant bacteria, discovery rate of carbapenem-resistant bacteria, antimicrobial drug use proportion (ADP), antimicrobial drug use intensity (ADI), carbapenem drug use proportion (CDPr), and carbapenem drug use percentage (CDPe). The detection rate and discovery rate of carbapenem-resistant bacteria were used to determine the presence of multidrug-resistant bacterial infections in the hospitals. ADP and ADI indicate the use and management of antibiotics in hospital inpatients. CDPr and CDPe indicate the use and management of carbapenem drugs specifically in inpatients.

### Pathogenic detection and drug sensitivity test

Carbapenem-resistant bacteria included carbapenem-resistant *Klebsiella pneumoniae* (CRKP), carbapenem-resistant *Escherichia coli* (CR-*E. coli*), carbapenem-resistant *Enterobacter cloacae* (CR-*E. cloacae*), CRAB and CRPA. CRE included CRKP, CR-*E. coli*, and CR-*E. cloacae*. Laboratory staff from each hospital conducted the bacterial culture, isolation, and verification of the samples submitted by the clinical departments. A Vitek 2-Compact (Merieux, France) and Phoenix 100 fully automated bacterial identification instrument (BD, USA) were used for bacterial identification. E-test strips (Bio-Merieux, France) were used to conduct drug sensitivity tests on the cultured pathogenic bacteria, and the drug sensitivity test results were analyzed in accordance with the American Association of Clinical and Laboratory Standards Antimicrobial Drug Sensitivity Test Implementation Standard M100 in 2020 (CLSI). The experimental strains were determined to be resistant to carbapenem antibiotics such as imipenem and meropenem. *Escherichia coli* (Clinical Laboratory Center of the National Health Commission) ATCC 25922 was used as a quality control strain.

### Different index calculation

The calculation was carried out according to the requirements of the “Quality Control Indices of Nosocomial Infection Management” and “Antibacterial Clinical Application Management Rating Indices” (2015 edition) issued by the Chinese National Health Commission. The calculation formula is as follows,

1. Detection rate of Carbapenem resistance bacteria: the detected number of strains of Carbapenem-resistant bacteria ÷ the total strains of the same bacterial species detected in the same period × 100%.
2. Discovery rate of carbapenem-resistant bacteria: the number of patients infected with carbapenem-resistant bacteria in hospitals (not including colonized strain infections) ÷ the total number of patients hospitalized in the same period × 100%.
3. ADP of inpatients: the number of inpatients who received antibiotics ÷ the total number of inpatients × 100%.
4. ADI of inpatients: the total DDD (defined daily dose) values of antibiotic consumption by inpatients ÷ number of days of patients admitted in the same period × 100%.
5. CDPr of inpatients: the number of inpatients who received carbapenems ÷ the total number of inpatients with antibiotic use in the same period × 100%.
6. CDPe: DDD of carbapenem use in 2019 ÷ DDD of antibiotics use in 2019 × 100%.

### Classification of different hospital levels and economic hospitals

Hospitals were divided into provincial and prefecture-level hospitals according to their administrative level. There were 11 provincial hospitals (H2, H3, H4, H6, H7, H8, H13, H15, H16, H21, and H28) and 25 prefecture-level hospitals (H1, H5, H9, H10, H11, H12, H14, H17, H18, H19, H20, H22, H23, H24, H25, H26, H27, H29, H30, H31, H32, H33, H34, H35, and H36). According to the per capita GDP of cities within the jurisdiction of Shandong Province, the hospitals were divided into three grades: high, middle, and low. A total of 19 hospitals (H1, H2, H3, H4, H5, H6, H7, H8, H9, H10, H11, H12, H13, H14, H15, H19, H27, H30, and H31) belonged to the high-economic-level, 11 hospitals (H16, H17, H21, H22, H25, H28, H29, H33, H34, H35, and H36) belonged to the medium economic level, and six hospitals (H18, H20, H23, H24, H26, and H32) belonged to the low economic level.

### Statistical analysis

Data were recorded by two experienced investigators to ensure accuracy. Additionally, uncertain or incomplete data were eliminated for data validity. The correlations between the detection rate of drug-resistant bacteria and different indices of antimicrobial drug usage were analyzed using the Spearman rank correlation method. The differences in detection rates across different hospital groups were examined using Kruskal–Wallis tests (several variables were non-normally distributed). All graphing and analyses were performed using R 3.5.0 (R Core Team, R Foundation for Statistical Computing, Vienna, Austria). A two-tailed *p-*value of <0.05 was considered statistically significant.

## Results

### Distribution of detection and discovery rates

**Figure 2:**
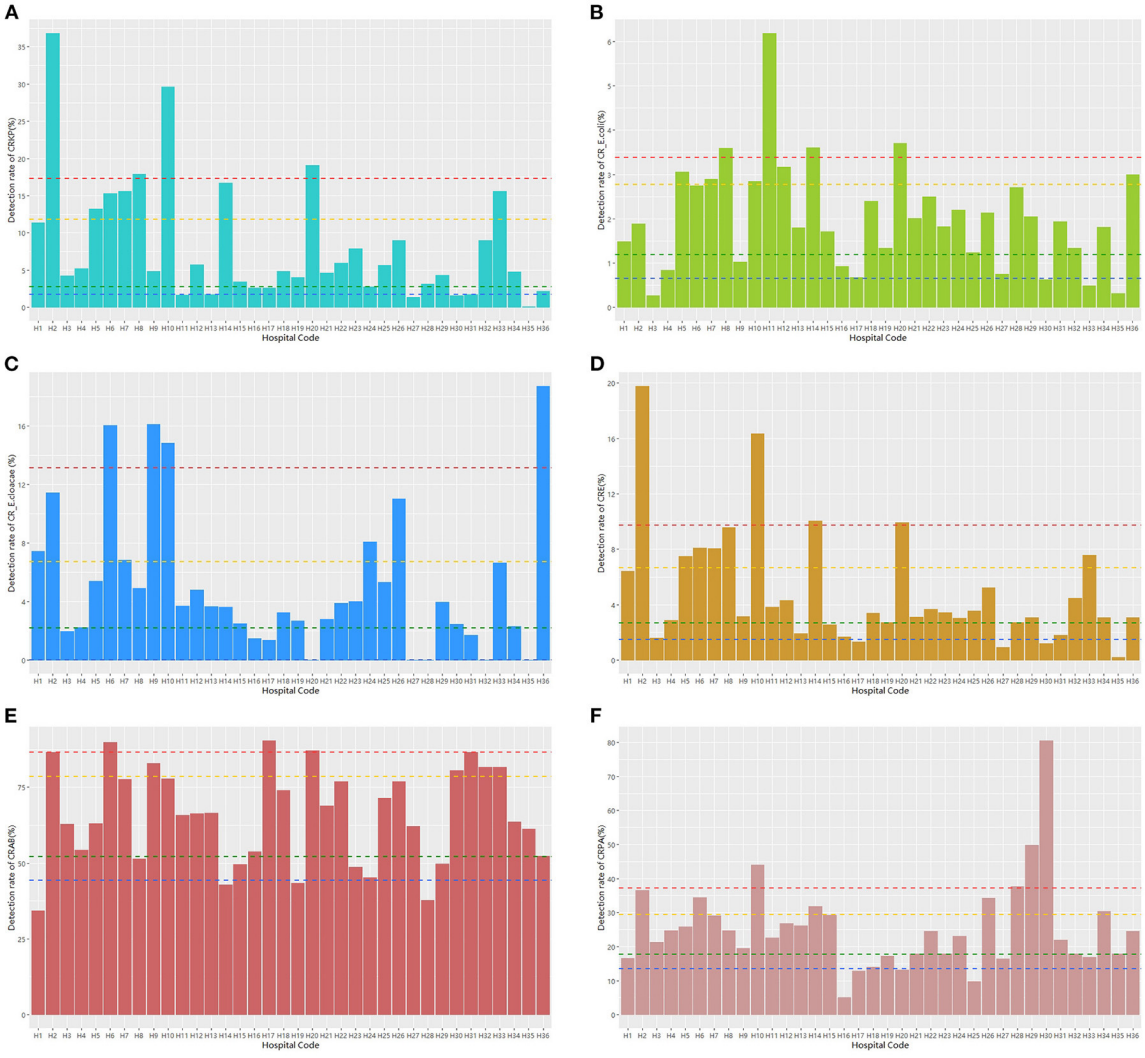
The detection rate distribution of different carbapenem-resistant bacteria. Notes: blue dashed line: the 10th percentile; green dashed line: the 25th percentile; yellow dashed line: the 75th percentile; and red dashed line: the 90th percentile.

The 50th percentile detection rate of CRE, CRAB, and CRPA were 3.28%, 66.04% and 23.80%, respectively. The detection rate of CRKP was above the 90th percentile (17.28%) in four hospitals, including H2, H8, H10, and H20, and that of nine hospitals was above the 75th percentile (11.83%), including H2, H5, H6, H7, H8, H10, H14, H20, and H33 (Figure 2A). The detection rate of CR-*E. coli* was above the 90th percentile (3.38%) in 4 hospitals, including H8, H11, H14, and H20, and that of nine hospitals was above the 75th percentile (2.77%), including H5, H7, H8, H10, H11, H12, H14, H20, and H36 (Figure 2B). The detection rate of CR-*E. cloacae* was above the 90th percentile (13.14%) in four hospitals, including H6, H9, H10, and H36, and that of nine hospitals was above the 75th percentile (6.71%), including H1, H2, H6, H7, H9, H10, H24, H26, and H36 (Figure 2C). The detection rate of CRE was above the 90th percentile (9.75%) in four hospitals, including H2, H10, H14, and H20, and that of nine hospitals was above the 75th percentile (6.67%), including H2, H5, H6, H7, H8, H10, H14, H20, and H33 (Figure 2D). The detection rate of CRAB was above the 90th percentile (86.51%) in three hospitals, including H6, H17, and H20, and that of nine hospitals was above the 75th percentile (78.50%), including H2, H6, H9, H17, H20, H30, H31, H32, and H33 (Figure 2E). The detection rate of CRPA was above the 90th percentile (37.13%) in four hospitals, including H10, H28, H29, and H30, and that of nine hospitals was above the 75th percentile (29.47%), including H2, H6, H10, H14, H26, H28, H29, H30, and H33 (Figure 2F).

In the 36 hospitals in Shandong, the overall discovery rates of CRKP, CR-*E. coli*, CR-*E. cloacae*, and CRE remained below 5 ‰, and the discovery rate of CRAB was below 10 ‰. Among the 36 hospitals, two hospitals (H7 and H13) had a high discovery rate for CRKP (Supplementary Figure 1A). High discovery rates for CR-*E. coli* and CR-*E. cloacae* were found at H7, H13, and H14 (Supplementary Figures 1B,C). Only H7 had high discovery rates of CRE, CRAB, and CRPA (Supplementary Figures 1D–F).

### The correlation of different indices

**Figure 3:**
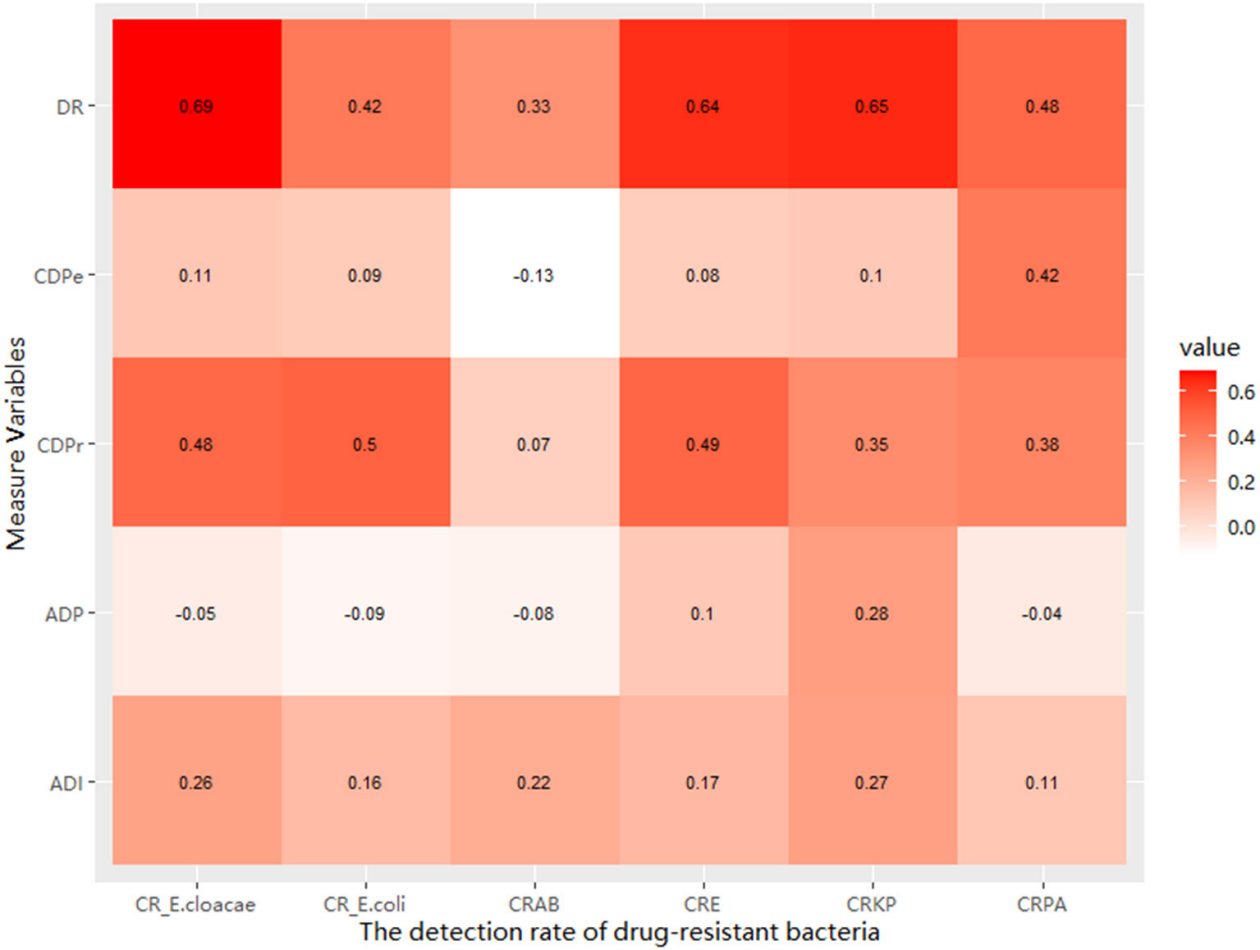
The relationship between the detection rate of GNRB and the discovery rate and the measured drug use variables.

In this study, the detection rate of drug-resistant bacteria showed a strong positive correlation with the discovery rate, and the correlation coefficient ranged from 0.33 to 0.69. Moreover, there was a positive association between the detection rate of drug-resistant bacteria and the intensity of antimicrobial drugs used. In addition, there was a positive association between the detection rate of drug-resistant bacteria (except CRAB) and discovery rate of drug-resistant bacteria and the proportion of patients who received carbapenems (Figure 3).

### Comparison based on hospital rank

**Figure 4:**
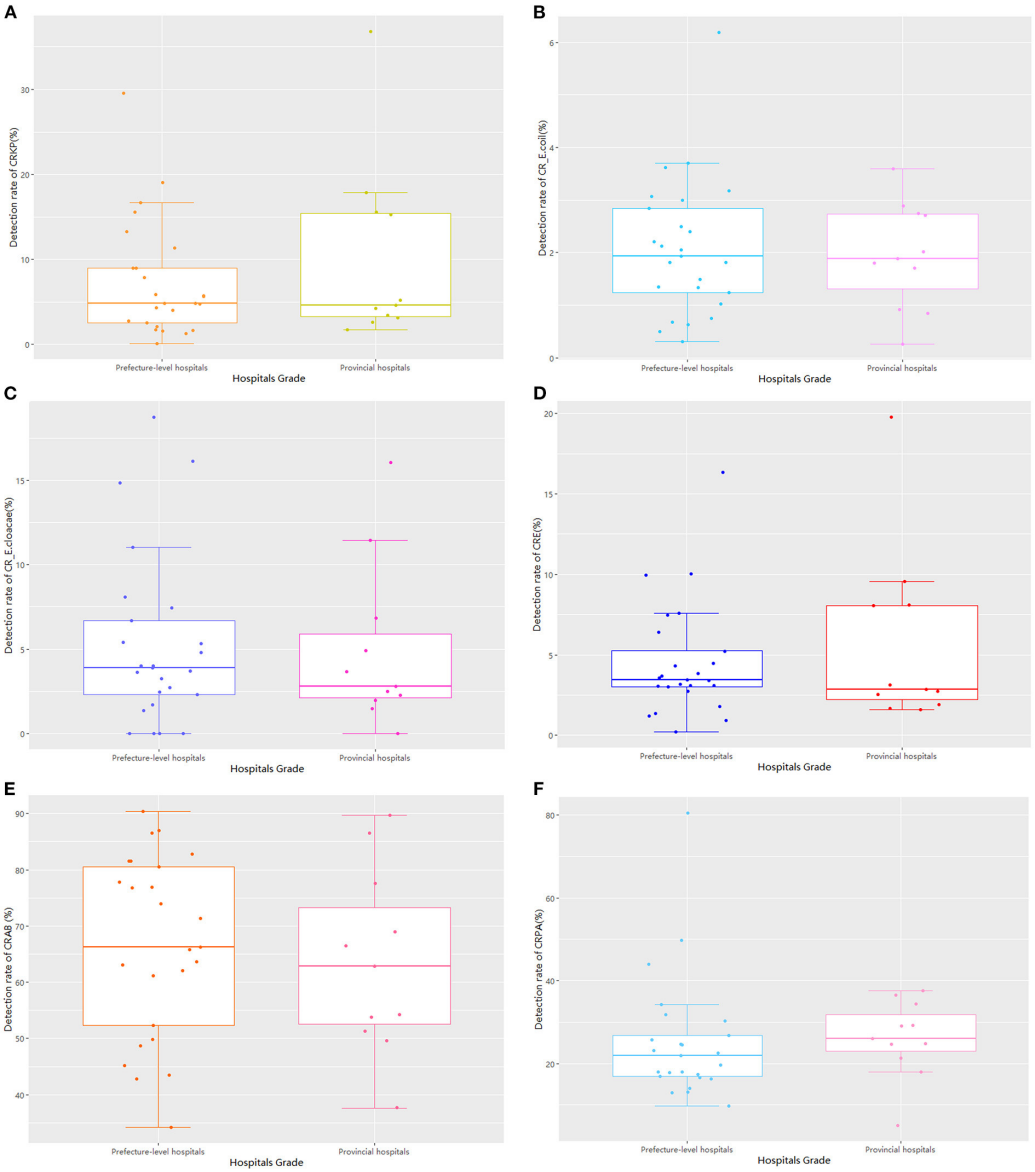
The comparison of detection rates of GNRB based on hospital administrative levels.

**Figure 5:**
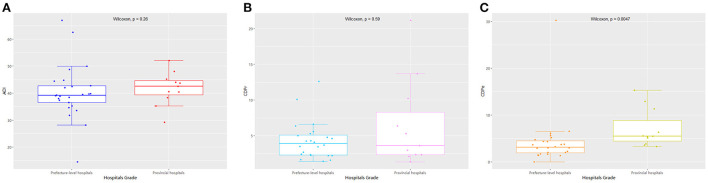
The comparison of the measured drug use indices of GNRB at different hospital levels.

Regarding hospital rank, there were 25 prefecture-level and 11 provincial hospitals, with no statistically significant differences between the two groups in the detection rate of drug-resistant bacteria (Table 1; Figure 4). The median and quartile of ADI, CDPr, and CDPe of prefecture-level and provincial hospitals were 39.2 (6.25), 42.5 (5.28); 3.89 (2.76), 3.61(5.96); and 3.13 (2.57), 5.51(4.38), respectively. The CDPe was significantly different between the prefecture-level and provincial hospitals (*P* < 0.05) (Figure 5).

**Table 1:** The detection rate of GRNB from different hospitals rank group.

	**Hospitals rank median (IQR)**		***P-*value**
**Detection rate (%)**	**Prefecture-level hospital (*n* = 25)**	**Provincial hospital (*n* = 11)**	
CRKP	4.84 (6.44)	4.60 (12.1)	0.68
CR_E.coil	1.93 (1.60)	1.88 (1.42)	0.87
CR_E.cloacae	3.90 (4.36)	2.81 (3.76)	0.73
CRE	3.43 (2.22)	2.87 (5.85)	0.84
CRAB	66.3 (28.2)	62.9 (20.7)	0.67
CRPA	21.9 (9.95)	26.1 (8.86)	0.18

### Comparison based on different hospital levels according to economic region

**Figure 6:**
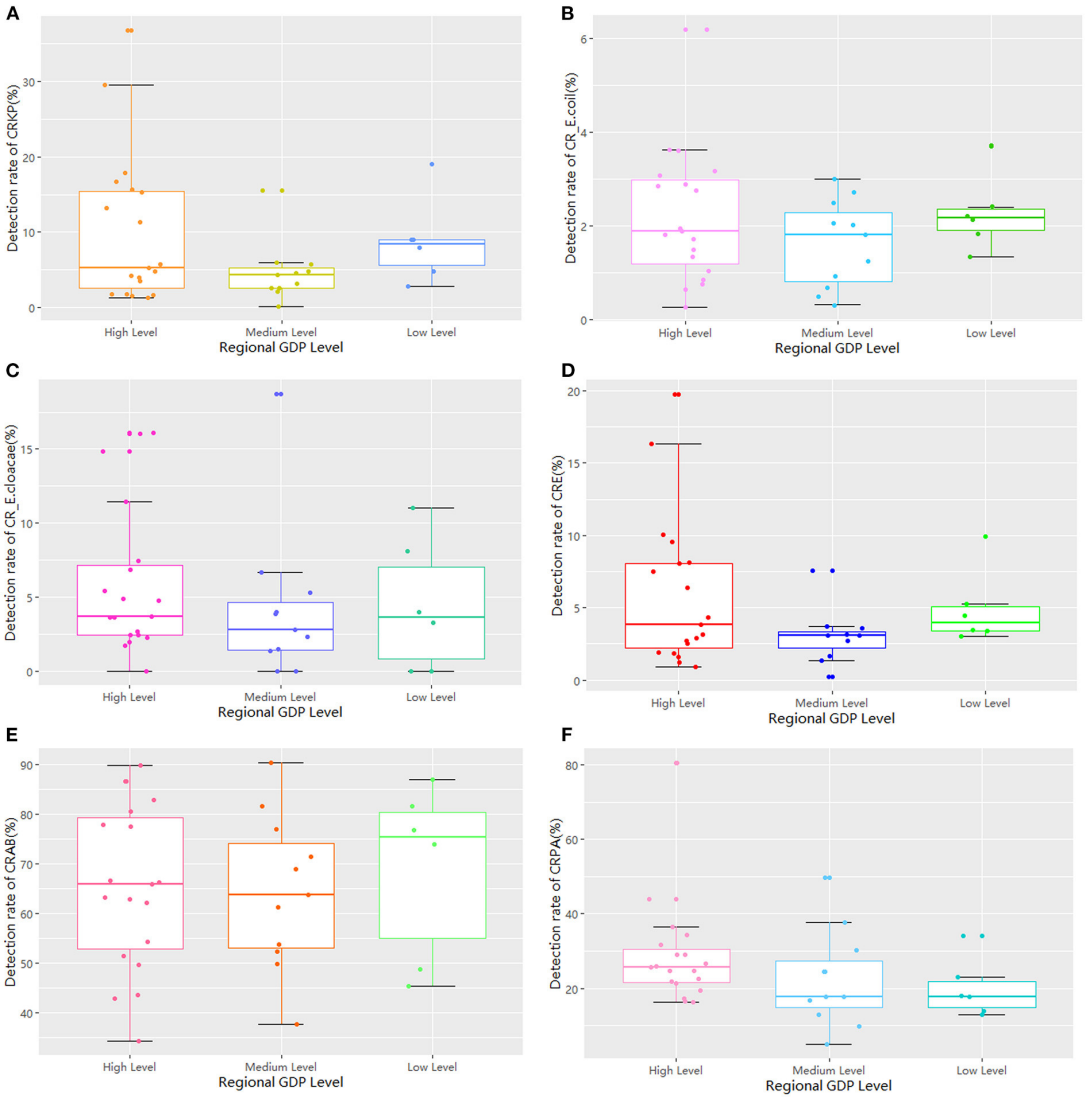
The comparison of detection rates of GRNB based on regional per capita GDP.

**Figure 7:**
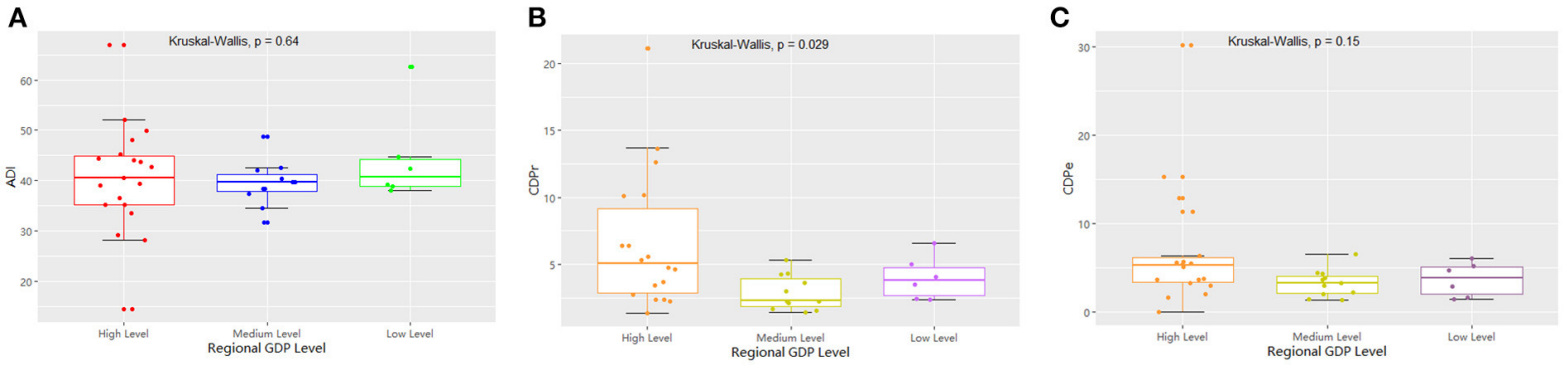
The comparison of the measured drug used indices of GRNB based on regional per capita GDP.

The 36 hospitals surveyed in the Province were divided into three groups for comparison based on the per capita GDP of their region. The results showed that there were 19 hospitals in regions with high economic status, 11 in regions with medium economic status, and six in regions with low economic status. There was no significant difference in the detection rate of drug-resistant bacteria between the three groups (Table 2; Figure 6). The median and quartile of ADI, CDPr, and CDPe were 40.6 (9.58), 39.7 (3.36), 40.8 (5.24); 5.27 (2.81), 3.30 (1.95), 3.85 (3.10); and 5.04 (6.28), 2.26 (2.06), 3.78 (2.08), respectively. Group comparisons showed that carbapenem use in hospitals in high economic regions was significantly higher than in hospitals in low economic regions (*P* < 0.05) (Figure 7).

**Table 2:** The detection rate of GRNB from different hospitals economic level group.

	**Hospitals rank median (IQR)**			***P*-value**
**Detection rate (%)**	**High level (*n* = 19)**	**Medium level (*n* = 11)**	**Low level (*n* = 6)**	
CRKP	5.22 (12.8)	4.34 (2.63)	8.45 (3.40)	0.23
CR_E.coil	1.88 (1.80)	1.81 (1.47)	2.16 (0.45)	0.37
CR_E.cloacae	3.70 (4.67)	2.81 (3.23)	3.63 (6.25)	0.50
CRE	3.84 (5.85)	3.07 (1.15)	3.95 (1.64)	0.22
CRAB	65.80 (26.40)	63.60 (21.10)	75.40 (25.40)	0.88
CRPA	25.80 (8.88)	17.90 (12.60)	17.90 (6.84)	0.13

## Discussion

Antibacterial resistance is a global threat to public health. The surveillance of bacterial resistance is one of the critical tasks mentioned in the action plan for tackling antibacterial resistance (12, 15, 16). In our study, we first surveyed the detection rate of CRGN and analyzed its association with antibiotic use in the hospitals included in this study. We found average detection rates of CRE, which was slightly higher than that reported by the China Antimicrobial Resistance Surveillance System report (17). In addition, the average detection rates of CRAB and CRPA in the investigated hospitals which were higher than the averages of 56% and 19.1%, respectively, in the China national report (15, 18). Compared other countries in world, the average detection rate of CRKP, CRAB and CRPA in Shandong Province of China were lower than the average of Southern European countries (Bulgaria, Italy, Greece and Romania), but it was higher than Northern and Western of Southern European countries (https://atlas.ecdc.europa.eu/). Moreover, the discovery rate in different hospitals was generally maintained at low levels, except for in few hospitals which indicated high nosocomial infection in patients. These findings suggest that CRGN still needs to be controlled in some hospitals in Shandong Province and effective measures need to be implemented.

Moreover, the higher detection rate of CRGN in different hospitals was associated with higher discovery rate and higher antimicrobial use frequency. There was a positive association between the detection rate of drug-resistant bacteria (except CRAB) and CDPr. Previous studies have shown that hospitalization and cumulative antibiotic exposure history, especially previous use of beta-lactams and carbapenems, are considered risk factors for CRGN infections (2–4, 8). Carbapenems have become the best and last-measure drug for treating these strains (6, 11). However, the frequent use of carbapenems has contributed to an increase in carbapenem resistance in Enterobacteriaceae (10). Additionally, the incidence of antimicrobial-resistant *A. baumannii* is high in mainland China. CRAB poses a significant threat to hospitalized patients, as therapeutic options are scarce. Alarmingly, the rates of carbapenem resistance in *A. baumannii* are on the rise and multi-drug resistance is becoming an increasingly common phenotype in this bacteria (1, 19–21).

We found no statistically significant difference in the detection rate of drug-resistant bacteria between prefecture-level and provincial hospitals. However, the CDPe of provincial hospitals was higher than that of prefecture-level hospitals. In addition, there was no statistically significant difference in detection rate among the three groups based on per capita GDP. However, the proportion of carbapenems used in high economic region hospitals was significantly higher than that of low economic region hospitals. These results suggest that there may be more critical cases in provincial and high economic region hospitals than in prefecture-level and low-economic region hospitals because provincial hospitals in high economic regions had better access to treatments, which led to increased carbapenem drug use. Furthermore, CRE infections are mainly an issue for inpatient facilities, where they are associated with mortality rates as high as 40–50%, and these resistant strains continue to increase in prevalence based on national reports. Interventions to curtail the spread of CRE in healthcare facilities have mostly involved bundled infection control measures (7, 22). Based on previous studies, successful solutions may include patient cohorts, contact isolation, dedicated staff, daily bathing of all patients with chlorhexidine, educating and training staff, limiting the use of invasive devices, shortening the duration of mechanical ventilation, improving hand hygiene rates and antimicrobial stewardship, and/or enhancing environmental cleaning.

Potential interventions in US facilities where CRE rates are still low include screening high-risk patients for CRE status on admission, such as for patients transferred from long-term care facilities. While awaiting screening results, hospitals may use preemptive contact precautions for such admissions, especially if rates are high in the referring facilities (3, 5, 23).

This study had several limitations. First, variations in detection platforms and technical skills may exist between hospitals, although the study itself had good external quality control. Second, we conducted a cross-sectional survey and lacked a longitudinal study design. In the future, implementing proper management strategies and reducing the unnecessary use of antibacterial drugs may be an effective measure to reduce the spread of CRGN, which should be confirmed by further studies. Bundled infection control measures, education and training, interventions aimed at healthcare-associated risk factors for colonization and/or infection, and proactive assessment of emerging community reservoirs may help thwart the rapid spread of these pathogens.

## Conclusion

These results report the general situation of CRGN in Shandong Province and revealed significant correlations between the use of carbapenems and resistance rates in gram-negative bacteria. Overall, hospital-level bacterial resistance surveillance is needed as part of antimicrobial stewardship measures in China, as local antimicrobial resistance data are critical for guiding the rational use of antimicrobials.

## Data availability statement

The original contributions presented in the study are included in the article/Supplementary material, further inquiries can be directed to the corresponding authors.

## Author contributions

KL and WL designed the study. HX and JS collected the data. KL analyzed the data. KL, WL, HX, JS, and YL wrote the paper. All authors contributed to the article and approved the submitted version.

## Funding

This work was in part supported by grants from the National Science and Technology Major Project of China (Grant No: 2018ZX10733402) and Major Scientific and Technological Innovation Project in Shandong Province (Grant No: 2019JZZY010719). The funding agency had no role in the study design, data collection and analysis, writing of this manuscript, or in the decision to submit the work for publication.

## Conflict of interest

The authors declare that the research was conducted in the absence of any commercial or financial relationships that could be construed as a potential conflict of interest.

## Publisher's note

All claims expressed in this article are solely those of the authors and do not necessarily represent those of their affiliated organizations, or those of the publisher, the editors and the reviewers. Any product that may be evaluated in this article, or claim that may be made by its manufacturer, is not guaranteed or endorsed by the publisher.

## Supplementary material

Supplementary Figure 1 The discovery rate distribution of different carbapenem-resistant bacteria.

## Abbreviations

CRGN: carbapenem-resistant gram-negative bacteria
CRE: carbapenem-resistant *Enterobacteriaceae*
CRAB: carbapenem-resistant *Acinetobacter baumannii*
CRPA: carbapenem-resistant *Pseudomonas aeruginosa*
CRKP: carbapenem-resistant *Klebsiella pneumoniae*
CR-*E. coli*: carbapenem-resistant *Escherichia coli*
CR-*E. cloacae*: carbapenem-resistant *Enterobacter cloacae*
DR: discovery rate.
