# Supplementary figures and images for: Investigation and analysis of carbapenem-resistant gram-negative bacterial infection rates across hospitals in Shandong Province in China
Source: Front Public Health. 2022 Nov 7;10:1014995. doi: 10.3389/fpubh.2022.1014995 (PMC9677124; doi:10.3389/fpubh.2022.1014995)

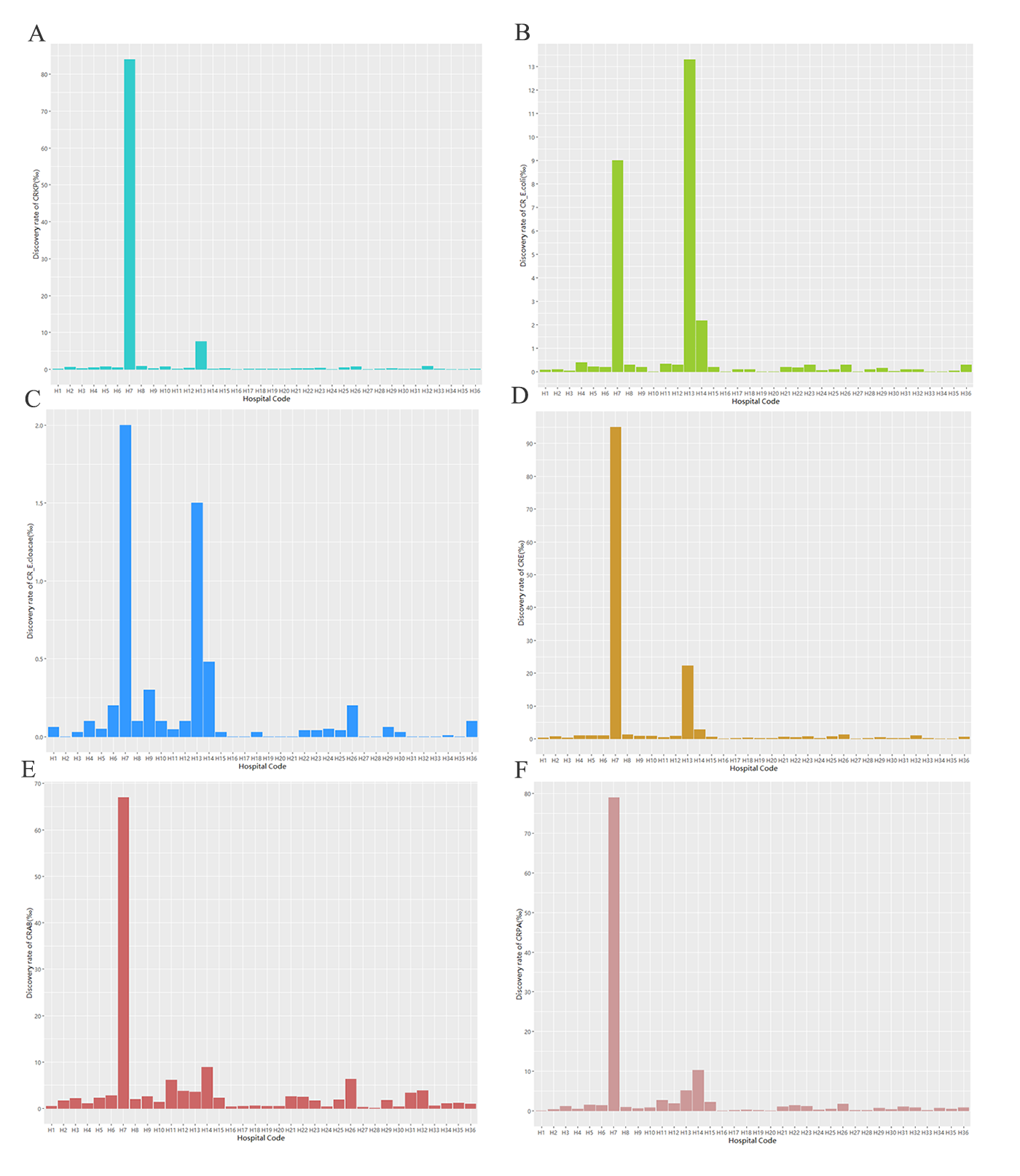

Supplement: Supplementary Figure 1 — The discovery rate distribution of different carbapenem-resistant bacteria. [file Image_1.tif]
